# Dengue Virus and the Host Immune System: A Battle of Immune Modulation, Response and Evasion

**DOI:** 10.3390/pathogens14111132

**Published:** 2025-11-07

**Authors:** Anwesha Ghosh, Sudipta Mondal, Soumyodip Sadhukhan, Provash Chandra Sadhukhan

**Affiliations:** Indian Council of Medical Research-National Institute for Research in Bacterial Infections, P-33, Scheme XM, CIT Road, Beliaghata, Kolkata 700010, West Bengal, India; anwesha.zoology@gmail.com (A.G.); msudipta99@gmail.com (S.M.); soumyodipsadhukhan2000@gmail.com (S.S.)

**Keywords:** dengue virus (DENV), immune evasion, host-pathogen interaction, antibody-dependent enhancement (ADE), severe dengue

## Abstract

Dengue virus (DENV) is a major global health concern, with pathogenesis driven by complex interactions between the virus, host genetics, and immune responses. Key determinants of disease severity include antibody-dependent enhancement (ADE), cross-reactive T cells, anti-NS1 antibodies, autoimmunity, and genetic predisposition, with the NS1 protein and its antibodies strongly implicated in severe dengue. This review highlights recent advances in our understanding of how DENV impacts host immune responses at cellular, molecular, and genetic levels. We particularly focus on how the virus interacts with the host, alters immune responses, and escapes immune detection. These factors are crucial for disease progression and immune dysfunction. The host mounts both innate and adaptive immune responses involving interferon signalling, cytokine production, antigen presentation, and T-cell activation. However, DENV evades immunity by suppressing interferon pathways, disrupting antigen presentation, and leveraging antibody-dependent enhancement (ADE), leading to immune dysregulation, prolonged viremia, and severe dengue. Gaining insight into these host-pathogen interactions is essential for understanding dengue pathogenesis for designing safer and more effective therapeutics. Furthermore, integrating omics approaches with immune response models shows promise for identifying early, reliable markers that can predict disease severity and guide treatment. A deeper understanding of these processes will support the development of personalised treatment strategies and enhance preparedness for future dengue outbreaks.

## 1. Introduction

Dengue virus (DENV) is one of the most significant vector-borne viral threats to global public health. According to the World Health Organization (WHO), the worldwide dengue burden reached unprecedented levels in 2024, with more than 7.6 million reported cases, over 3.4 million laboratory-confirmed infections, and over 3000 deaths across endemic regions [1]. Endemic in more than 100 countries, mainly in tropical and subtropical zones, dengue affects an estimated 390 million people each year, of whom nearly 96 million develop clinical symptoms [2]. Factors such as rapid urbanisation, climate change, and inadequate vector control have collectively driven the resurgence and geographical spread of dengue, making it a vital global health concern.

DENV circulates in nature as four antigenically distinct serotypes (DENV-1 to DENV-4), each capable of causing a range of diseases from mild febrile illness to severe conditions such as severe dengue [3]. Primary infection with one serotype usually results in long-lasting immunity against that serotype; however, this protection does not strongly extend to heterologous serotypes. Conversely, secondary infection with a different serotype can lead to a paradoxical increase in disease severity, a phenomenon known as antibody-dependent enhancement (ADE) [4]. In ADE, pre-existing non-neutralising antibodies aid the virus in entering Fc receptor-bearing immune cells, thereby increasing viral replication and triggering hyperinflammatory responses [5].

DENV is a positive sense RNA virus with a genome size of 10.7 kb with three structural proteins [capsid, pre-membrane/membrane (prM/M), envelope (E)] and seven nonstructural proteins (NS1, NS2A, NS2B, NS3, NS4A, NS4B, NS5). NS1 plays multiple roles in the DENV life cycle, including participation in RNA replication and evasion of the complement pathway. In addition, accumulating evidence shows that DENV NS1 contributes to pathogenesis by disrupting vascular integrity and modulating host immune responses. NS1 can directly damage endothelial cells by activating cathepsin L, heparanase, and sialidases, leading to degradation of the glycocalyx and disruption of intercellular junctions, which results in increased permeability. It also stimulates TLR4-expressing immune cells to produce proinflammatory cytokines and vasoactive mediators that further compromise endothelial barrier function. Moreover, NS1 interferes with the complement system by activating or degrading its components, thereby protecting the virus from immune clearance and enhancing viral replication [6,7].

The initial antiviral defence is mediated by the innate immune system, where pattern recognition receptors such as Toll-like receptors (TLRs), RIG-I, and MDA-5 detect viral RNA and activate interferon signalling pathways [8]. However, DENV has evolved mechanisms to subvert these responses, such as the proteasomal degradation of STAT2 to dampen type I interferon signalling, thereby evading early immune clearance [9]. Infected innate immune cells, including monocytes, dendritic cells, and macrophages, become sources of excessive cytokine production (e.g., IL-6, TNF-α), contributing to vascular leakage and systemic inflammation [10].

The adaptive immune response plays a dual role in controlling infection and contributing to disease development. While CD8^+^ cytotoxic T lymphocytes (CTLs) and CD4^+^ helper T cells are essential for clearing viruses, faulty T cell responses, especially during secondary infections, can cause a cytokine storm and damage tissues [11]. Moreover, cross-reactive memory T cells with suboptimal affinity may exacerbate immunopathology, causing severe illness [12].

Host genetic makeup markedly influences susceptibility to severe dengue outcomes. Variants in genes encoding cytokines, HLA alleles, and pattern recognition receptors have been linked to varied immune responses and disease severity across different populations [13]. For example, polymorphisms in TNF-α, DC-SIGN, and MICB genes are known to impact immune activation thresholds and viral clearance efficiency [14]. Understanding the interaction between host genomics and immune regulation is therefore vital for risk stratification, vaccine development, and designing host-targeted therapeutics.

This review highlights recent advances in understanding how the DENV interacts with host immune components at the cellular, molecular, and genetic levels, with a particular focus on mechanisms of host interaction, immune modulation, and immune escape that influence disease pathogenesis and clinical outcomes. Elucidating these host-pathogen dynamics is essential not only for clarifying dengue pathogenesis but also for guiding the development of next-generation vaccines, immunotherapies with broader efficacy and safety profiles, and the identification of reliable biomarkers for early diagnosis and disease severity prediction.

## 2. Structure of DENV

DENV, a small yet structurally complex pathogen, belongs to the *Flaviviridae* family and measures about 50 nanometres in diameter. Encased by a lipid bilayer derived from the host, the virus displays icosahedral symmetry, a shape that conceals the complex molecular interactions beneath its smooth surface [15].

**Figure 1:**
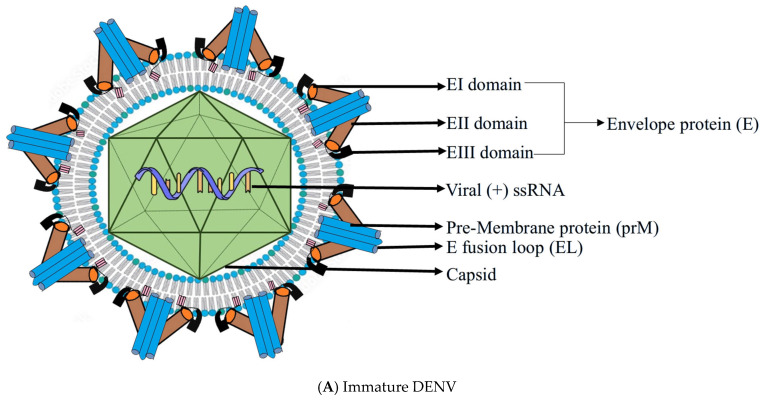

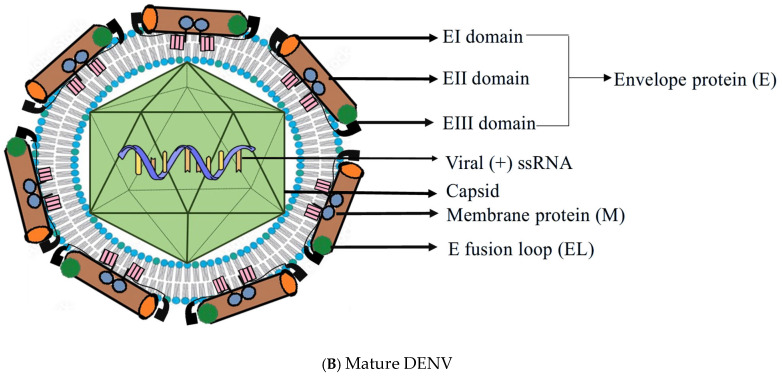
(**A**,**B**): Structural comparison between immature and mature DENV. (**A**) shows the immature DENV structure, characterized by the presence of pre-membrane proteins (prM) associated with envelope (E) proteins, which prevent premature fusion during viral assembly. The E proteins have three domains (EI, EII, EIII) and contain the fusion loop (EL). Inside, the capsid encloses the viral (+) ssRNA genome. (**B**) shows the mature DENV structure, where prM is cleaved to membrane protein (M) during viral maturation, allowing E proteins to rearrange into a smooth surface conformation. The capsid still encloses the viral RNA, and the E fusion loop (EL) becomes exposed, facilitating host cell entry [19].

The viral genome is a single-stranded, positive-sense RNA roughly 10.7 kilobases in length. It encodes a single polyprotein that is enzymatically cleaved into three structural proteins—capsid (C), precursor membrane (prM), and envelope (E)—along with seven non-structural proteins necessary for replication and immune modulation [3]. The envelope (E) protein dominates the viral surface and serves as the primary target for host immune recognition. It is divided into three functional domains: EDI (central core), EDII (containing the fusion loop), and EDIII (involved in receptor binding). (Figure 1) During infection, E proteins transition from a dimeric to a trimeric form, a conformational change essential for fusion with host membranes. This dynamic process, termed “viral breathing,” exposes cryptic epitopes and plays a key role in immune evasion, vaccine development, and antibody neutralization [16].

Beneath the envelope lies the prM protein, a chaperone-like structure that stabilizes E during viral assembly. As the virion matures through the trans-Golgi network, prM is cleaved by the host protease furin into M protein. However, incomplete cleavage often results in partially mature virions that maintain infectivity and alter antibody accessibility, contributing to antibody-dependent enhancement (ADE) during secondary infections [16]. The innermost component, the capsid (C) protein, binds and protects the viral RNA through electrostatic interactions. Interestingly, while the envelope demonstrates symmetrical arrangement, the nucleocapsid is structurally disordered, a feature thought to aid in efficient genome packaging and virion flexibility [17]. Advancements in cryo-electron microscopy and crystallography have further demonstrated how temperature and pH fluctuations influence virion conformation [18].

## 3. Replication Cycle of DENV

**Figure 2:**
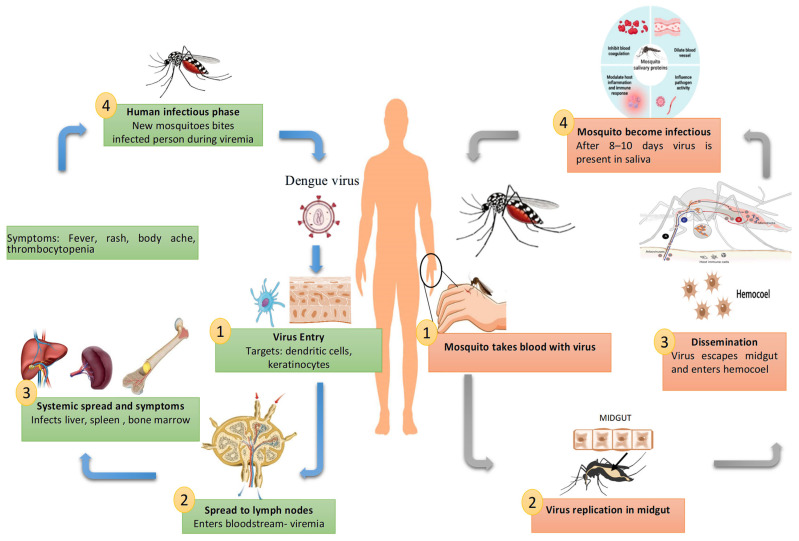
Transmission cycle and pathogenesis of DENV in humans and mosquitoes. The figure illustrates the DENV life cycle between humans and mosquitoes. In humans, the virus first enters skin cells, then spreads to lymph nodes and the bloodstream, leading to systemic infection in organs such as the liver, spleen, and bone marrow, and causing symptoms including fever, rash, body ache, and thrombocytopenia. During the viremic phase, mosquitoes acquire the virus when feeding on infected individuals. In mosquitoes, the virus replicates in the midgut, disseminates to the hemocoel, and reaches the salivary glands within 8–10 days, after which the mosquito becomes infectious and can transmit the virus to another human.

DENV is transmitted primarily by *Aedes aegypti* and *Aedes albopictus* mosquitoes. In mosquitoes, ingestion of viremic blood initiates viral replication in the midgut, followed by dissemination to the salivary glands, from where infectious virions are released during subsequent bites (Figure 2).

The replication cycle of the DENV is a carefully coordinated process that occurs within human host cells, skilfully navigating cellular machinery and immune responses. This cycle involves seven main stages: attachment, entry, fusion and uncoating, translation, replication, assembly, and release. Each step not only promotes viral propagation but also subtly influences host immunity and the severity of the disease [3].

DENV initiates infection by binding to various cell surface receptors. While dendritic cell-specific intercellular adhesion molecule-3-grabbing non-integrin (DC-SIGN) and heparan sulfate are commonly exploited, recent studies highlight the involvement of TIM and TAM family receptors, particularly in monocytes, macrophages, and endothelial cells [20]. After receptor engagement, the virus enters cells via clathrin-mediated endocytosis, forming endosomes. As endosomes acidify, the viral envelope (E) protein undergoes significant conformational changes, shifting from dimers to fusogenic trimers. This transition exposes a hydrophobic fusion loop, enabling fusion of the viral and endosomal membranes and releasing the RNA genome into the cytoplasm; a process critically dependent on pH and E protein dynamics [21]. Once inside, the positive-sense RNA genome is translated directly by host ribosomes into a single polyprotein. This precursor protein is cleaved by host and viral proteases into structural and non-structural components. The non-structural proteins, notably NS3 (helicase/protease) and NS5 (RNA-dependent RNA polymerase), orchestrate genome replication and immune modulation [22]. Viral replication occurs on virus-induced membranous platforms derived from the endoplasmic reticulum. These “replication factories” enable the synthesis of a negative-strand RNA intermediate, which then serves as a template to produce multiple positive-sense progeny genomes [17]. Notably, DENV hijacks host lipid metabolism and autophagic pathways to optimize this replicative niche [23].

**Figure 3:**
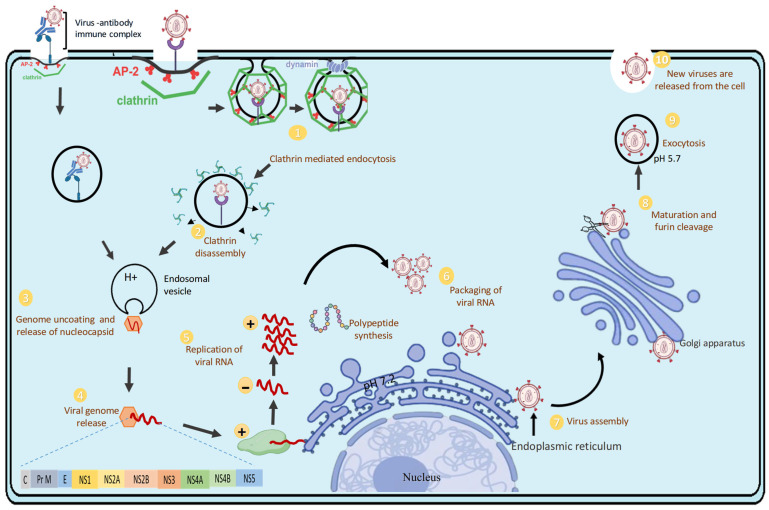
Replication cycle of DENV.

As illustrated in Figure 3, the DENV replication cycle begins when the virus or virus–antibody complex binds to host cell receptors or Fcγ receptors, facilitating entry via clathrin-mediated endocytosis (step 1). Once internalized, the clathrin coat is removed, forming an endosomal vesicle that carries the virus deeper into the cell (step 2). Acidification of the endosome (low pH) triggers conformational changes in the viral envelope proteins, leading to fusion of the viral and endosomal membranes and release of the nucleocapsid (step 3). The nucleocapsid disassembles, releasing the viral positive-sense single-stranded RNA (+ssRNA) into the cytoplasm (step 4). The viral RNA acts as mRNA for direct translation into a single polyprotein, which is processed into structural and non-structural proteins. Replication occurs on ER-derived membranes, producing both negative-sense RNA intermediates and new positive-sense genomes (step 5). Newly synthesized viral RNA is packaged with capsid proteins (step 6). Immature virions assemble in the endoplasmic reticulum (ER), incorporating prM and E proteins (step 7). Immature virions are transported through the Golgi apparatus, where the host enzyme furin cleaves prM into M, allowing for the structural rearrangements required for viral maturation (step 8). Mature virions are transported to the cell membrane in vesicles and released by exocytosis at a mildly acidic pH (~5.7) (step 9). Fully mature infectious dengue virions are released into the extracellular space, ready to infect new host cells (step 10) [3,24].

Following genome replication, the new RNA strands interact with capsid proteins to form nucleocapsids. These are enveloped by prM and E proteins as they bud into the ER lumen, forming immature virions. Maturation occurs as prM is cleaved by furin in the Golgi, a crucial step for rendering the virus infectious. However, incomplete cleavage produces a range of particles with different immunogenic profiles [24]. Finally, mature virions are transported via secretory vesicles and released through exocytosis to infect neighbouring cells or spread systemically, continuing the viral lifecycle and its immunological effect [25].

## 4. Host Immune Response Models and Immune Escape Mechanisms of DENV

**Figure 4:**
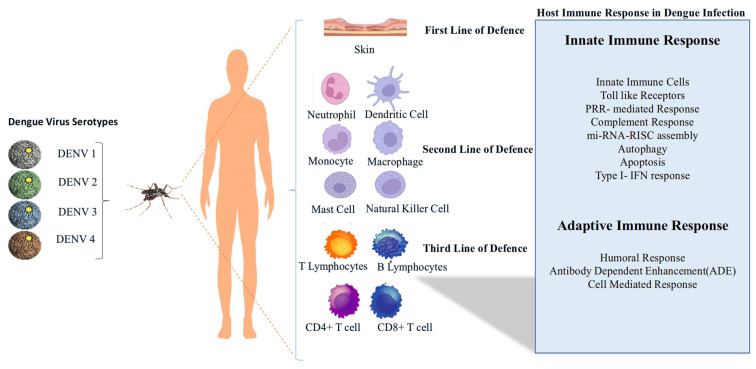
Host Immune Response in DENV infection.

The host immune response to DENV infection highlights its progression through three lines of defence. The virus, transmitted by mosquitoes, exists in four serotypes (DENV-1 to DENV-4). The first line of defence involves the skin and resident immune cells such as neutrophils and dendritic cells. The second line of defence includes monocytes, macrophages, mast cells, and natural killer cells. The third line of defence engages adaptive immunity, involving T and B lymphocytes, as well as CD4^+^ and CD8^+^ T cells (Figure 4) [26].

### 4.1. Innate Immune Response to DENV

#### 4.1.1. Initial Immunological Encounter: Skin Resident IMMUNE Cells and Keratinocytes

The initial layer of cells infected with the DENV includes mast cells, macrophages, and dendritic cells. Dendritic cells, macrophages, and Langerhans cells are well known for becoming infected with DENV, although there is disagreement about which skin layer shows more DENV-infected cells [27,28]. Some studies suggest that there are 100 times more DENV-infected cells in the dermis compared to the epidermis at 72 h post-inoculation [28]. Conversely, other research indicates that keratinocytes alone account for up to 60% of DENV-infected cells, and that the epidermis contains roughly six times as many DENV-infected cells as the dermis at 48 h post-infection [29]. Additionally, tissue-resident mast cells (MC) are activated by DENV infection, and these MCs, through degranulation, induce endothelial cell activation, which leads to clinical symptoms such as rash in the skin [30,31]. MCS also produce and releases CXCL2, promoting neutrophil activation [27]. Furthermore, MCs are permissive to DENV infection via antibody-dependent enhancement (ADE), causing the release of Type I IFN. These interferons provide essential signals that facilitate the activation and chemotactic recruitment of circulating monocytes, resident tissue macrophages, natural killer (NK) cells, and neutrophils [27].

#### 4.1.2. Subsequent Wave: Monocyte-Derived Immune Cells Are Recruited and Infected; Concurrent Activation of Plasmacytoid Dendritic Cells

Tissue-resident macrophages, mast cells, and other initially infected cells secrete chemokines that recruit local myeloid cells, monocyte-derived macrophages, monocyte-derived dendritic cells (moDCs, Ly6C^+^ CD11b^+^), and circulating plasmacytoid dendritic cells (pDCs, CD123^+^) into the skin [28]. CCL2, IL-1β, and CCL20 are responsible for recruiting myeloid cells; CCL1 and CCL5 facilitate the extravasation of monocytes, and CCL2 promotes the recruitment of plasmacytoid dendritic cells (pDCs) into the inflamed skin [32]. Studies suggest monocyte-derived dendritic cells are more susceptible to infection than macrophages, whereas pDCs are activated by other DENV-infected cells through contact-dependent mechanisms [33]. Once activated, they become the leading producers of Type I interferons. Evidence indicates that pDCs are vital for controlling the virus; a reduction in pDC numbers in the blood has been associated with a higher risk of severe dengue in children [34].

#### 4.1.3. PRR-Mediated Recognition and Response, Type-I IFN Response to DENV Infection

*Aedes aegypti* mosquitoes transmit DENV to humans through their bite, which then infects and replicates in Langerhans cells and keratinocytes. This initiates a variety of innate immune responses in the host. Immune cells such as dendritic cells (DC), monocytes, and macrophages respond to pathogen-associated molecular patterns using their pattern recognition receptors (PRRs). These PRRs include cytoplasmic retinoic acid-inducible gene I (RIG-I) and melanoma differentiation-associated gene 5 (MDA-5), as well as endosomal Toll-like receptors 3 (TLR3) and TLR7 [35]. Cells in the myeloid, epithelial, and central nervous systems contain RIG-like receptors (RLRs) in their cytoplasm. They detect phosphate-containing RNA and long double-stranded RNA (dsRNA), aiding in recognising viral replication. RLRs migrate to the mitochondrial membrane via caspase activation recruitment domains (CARD), leading to activation of TANK-binding kinase 1 (TBK1) and IκB kinase-ε (IKKε), which phosphorylate IRF3 and IRF7, prompting their entry into the nucleus to stimulate the production of type I interferons (IFNs) such as IFN-β. TLR3 recognises dsRNA by activating the TRIF (TIR-domain-containing adapter-inducing interferon-β) pathway [36]. IFNα/β bind to IFNα/β receptors (IFNAR) on the surface of infected cells, further activating the JAK-STAT pathway. This pathway forms a complex with IRF9, which induces interferon-stimulated genes (ISGs) and chemokines [37].

#### 4.1.4. Infection-Driven Activation and Homing of Natural Killer Cells and Neutrophils

Bone marrow-derived NK cells are recruited to infected organs and tissues in response to several chemokines, which are known for directing NK cell chemotaxis, as well as activating mast cells and monocytes. These chemokines include CCL2, CCL3, CCL4, CXCL2, CXCL12, and IFN [38]. NK cells are activated by cytokines through their cytokine receptors, and they also respond to signals from ligands expressed on host (target) cells via activating and inhibitory receptors. Evidence suggests significant activation of NK cells during acute DENV-2 infection, with the production of substantial IFN-γ by NK cells [39]. A previous study conducted in paediatric patients with severe dengue in Thailand reported a significantly increased frequency of circulating activated NK cells, characterised by the expression of CD56 and the activation marker CD69, compared to patients with milder forms of dengue [40]. Neutrophils, a type of phagocytic granulocyte, play a central role in controlling bacterial infections. Their activation is triggered by damage-associated molecular patterns (DAMPs), pathogen-associated molecular patterns (PAMPs), pro-inflammatory cytokines, and complement-derived fragments such as CXCL2, TNF, C5a, and C3a [41]. Although their role in viral infection is not fully understood, a few studies suggest that patients infected with DENV exhibit elevated levels of circulating neutrophils during infection, indicating neutrophil activation. More recent findings have shown that neutrophil elastase activity, a significant component of neutrophil granules—is markedly higher in DENV-infected individuals compared to healthy controls, with its levels positively correlating with disease severity [42].

**Figure 5:**
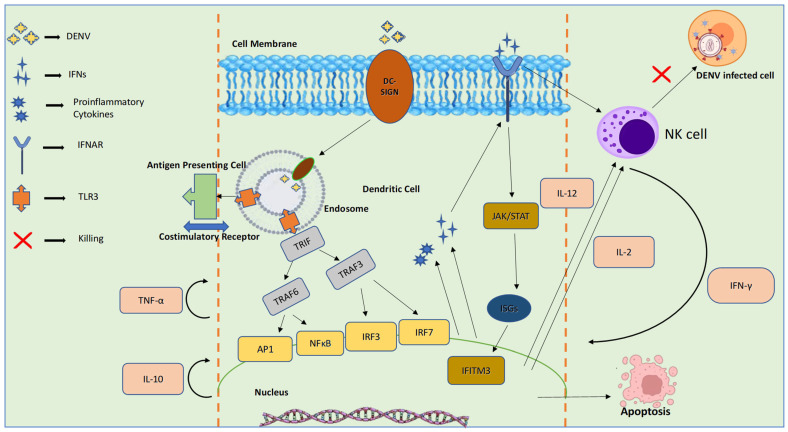
Innate Immune Signalling in DENV Infection.

Figure 5 demonstrates the innate immune signalling pathways activated during DENV infection, focusing on dendritic cell (DC) recognition and natural killer (NK) cell responses. DENV binds to dendritic cells via DC-SIGN and is internalized into endosomes, where pattern recognition receptors such as TLR3 detect viral components. This activates adaptor proteins (TRIF, TRAF3, TRAF6) and transcription factors (AP1, NFκB, IRF3, IRF7), leading to the production of proinflammatory cytokines (e.g., TNF-α, IL-10) and type I interferons (IFNs). IFNs stimulate interferon-stimulated genes (ISGs) like IFITM3 through the JAK/STAT pathway. Cytokines such as IL-12 and IL-2 activate NK cells, which produce IFN-γ and induce apoptosis of DENV-infected cells, thereby limiting viral spread [9].

### 4.2. Activation and Regulation of the Complement System in DENV Infection

The mannose-binding lectin pathway (MBL), among the complement pathways, is one of the primary factors contributing to innate immune response against viruses by recognising DENV through the MBL complex. This complex cleaves C4 and C2 via MBL-associated serine protease 2, producing C4b and C2b on the virion surface. This leads to the formation of C3 convertase, which further activates the classical complement cascade to form C5 convertase and ultimately the C5b-9 membrane attack complex (MAC), inducing viral lysis [36].

### 4.3. Role of miRNA-RISC Assembly in Viral RNA Silencing and Degradation of DENV

The small non-coding miRNA and siRNA play roles in antiviral immunity and mediate RNA interference. Key components of miRNA are Drosha and, in the case of siRNA, Dicer. These small RNAs guide the RNA-induced silencing complex (RISC) to target viral mRNA for degradation or translation inhibition via Argonaute (Argo) proteins, thereby suppressing viral replication, including DENV [43]. Various experimental studies have shown that silencing key RNAi components like Dicer, Drosha, Argonaute 1, and Argonaute 2 increases DENV replication in human cells, emphasising the antiviral role of RNAi [44]. One study demonstrated that miRNA-34 enhanced the production of type I interferons and the expression of interferon-stimulated genes (ISGs) by suppressing the Wnt-signalling pathway, thereby leading to reduced viral replication. Additional evidence from cell culture studies has shown that DENV infection induces the upregulation of miRNA-30e* in HeLa and U937 cells [45]. This upregulation helps restore type I interferon production and inhibits DENV replication by targeting IκBα, which in turn activates NF-κB signalling.

### 4.4. DENV and Autophagy

Autophagy is a process where cells form double-membrane vesicles called autophagosomes that engulf damaged organelles, misfolded proteins, or pathogens. These autophagosomes then fuse with lysosomes, where their contents are broken down and recycled. Evidence indicates that DENV infection activates the autophagy pathway; however, the role of autophagy in DENV infection varies depending on the cell type. In liver cells, autophagy exhibits pro-viral activity, with the DENV modifying the host cells’ lipid metabolism to generate the energy necessary for its optimal replication [23]. Of the DENV proteins, only NS4A and NS1 have been associated with autophagy. During infection, the pro-inflammatory cytokine macrophage migration inhibitory factor (MIF) released by liver, immune, and endothelial cells has been shown to promote shock by inducing autophagy-driven degradation of vascular endothelial cells, resulting in vascular leakage. In endothelial cells, autophagy uses the specialised receptor FAM134B to suppress DENV infection [46].

### 4.5. DENV and Apoptosis

Apoptosis is a regulated process of programmed cell death marked by specific structural and biochemical changes. Increasing evidence indicates that the DENV can induce apoptosis in host cells, and this response seems to vary depending on the cell type involved. During DENV infection, structural proteins such as the capsid protein (C) can trigger apoptosis by activating death-associated protein 6 and initiating Fas-mediated apoptotic pathways in liver cells. Conversely, non-structural proteins like the NS2B-NS3 protease precursor and the NS3 protease promote apoptosis via the caspase-8 cascade and the NF-κB signalling pathway [36]. Apoptosis has been observed in the liver, brain, and endothelial cells during autopsies of patients who suffered from severe dengue [47]. Evidence also suggests that inhibitors of apoptosis can delay DENV-induced cell death, creating a more favourable environment for viral replication. DENV sometimes activates P13K signalling early in infection, leading to the blockage of caspase-dependent apoptotic cell death [48]. This may be a mechanism of immune evasion in DENV-induced apoptosis.

### 4.6. Adaptive Immune Response

DENV glycoproteins are detected by the host immune system, which triggers immunological memory and forms the foundation of the adaptive immune response. The adaptive immune response includes both humoral (antibody-mediated) and cell-mediated immune responses.

#### 4.6.1. Humoral Immune Response

The phenomenon of original antigenic sin (OAS) influences the humoral immune response by causing the immune system to depend on memory from a previous, exposure of infection [49]. Although initially protective, this response becomes problematic when the new antigen differs substantially, resulting in an inadequate immune reaction. In DENV infections, OAS has been associated with more severe disease, as observed in Thai children who developed serious symptoms after a secondary infection with a different DENV serotype due to an unbalanced immune response [12].

Antibody-dependent enhancement (ADE) is an immune response mediated by antibodies and usually occurs during secondary infection with a different (heterotypic) DENV serotype. Evidence indicates that neutralising antibodies produced during a primary infection can offer adequate protection during a subsequent infection with the same (homotypic) serotype [50]. These neutralising antibodies mainly target epitopes on domain III of the envelope (E) protein, as well as the prM/M and NS1 proteins.

During primary DENV infection, such antibodies confer long-lasting immunity. However, in secondary infections involving a different serotype, these antibodies only offer short-term protection and partial immunity. In some cases, instead of neutralising the virus, they facilitate ADE by forming complexes with viral epitopes that promote viral entry into Fc receptor-expressing immune cells, leading to increased viral replication and more severe disease outcomes [51].

**Figure 6:**
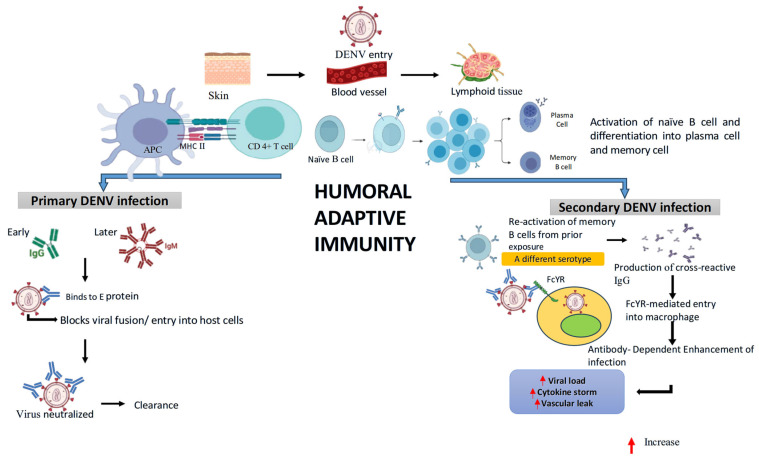
Humoral adaptive immune response during primary and secondary DENV infection. During primary DENV infection, antigen-presenting cells activate CD4^+^ T cells, which help naïve B cells differentiate into plasma cells and memory cells. These produce virus-specific antibodies (early IgM and later IgG) that neutralize the virus by blocking its entry into host cells. In secondary infection with a different DENV serotype, memory B cells produce cross-reactive IgG. Instead of neutralizing, these antibodies facilitate viral entry into macrophages via Fcγ receptors. This antibody-dependent enhancement (ADE) increases viral load, triggers cytokine storms, and may cause severe complications like vascular leakage, contributing to more severe disease outcomes.

As illustrated in Figure 6, during primary infection, DENV enters via the skin and is presented by antigen-presenting cells (APCs) through MHC II to CD4^+^ T cells, which help activate naïve B cells in lymphoid tissue. Activated B cells differentiate into plasma cells and memory B cells. Early IgM and later IgG antibodies bind to the viral E protein, blocking viral fusion and entry into host cells, leading to virus neutralization and clearance. In secondary infection, when a different DENV serotype infects, memory B cells from prior exposure are reactivated. This produces cross-reactive IgG that binds the virus but facilitates Fcγ receptor (FcγR)-mediated entry into macrophages, a process known as antibody-dependent enhancement (ADE). ADE increases viral load, triggers cytokine storms, and promotes vascular leakage, contributing to severe disease outcomes.

Scientific evidence supporting ADE has been shown in both animal models and human studies. Experiments with passively immunised non-human primates and mice have demonstrated that ADE can increase viral load and turn otherwise non-lethal DENV infections into fatal ones. Similar results have also been observed in human clinical cases. Collectively, these studies indicate that ADE can drive the progression of a usually mild and self-limiting DENV infection into a more severe and potentially life-threatening form [52].

#### 4.6.2. Cell-Mediated Immune Response

Cell-mediated immunity (CMI) is a part of the adaptive immune response, involving various subsets of T cells. CD8^+^ T cells identify and eliminate DENV-infected cells by recognising viral peptides displayed on MHC class I molecules. These CTLs release cytolytic granules containing perforin and granzymes, and secrete antiviral cytokines such as IFN-γ, TNF-α, IL-2, and GM-CSF, as well as chemokines like CCL3 (MIP-1α), CCL4 (MIP-1β), and CCL5 (RANTES), which collectively enhance immune cell recruitment, activate macrophages, and restrict viral replication. Previous studies have demonstrated that CD8^+^ T cell responses to mainly targeted non-structural proteins, notably NS3 and NS5, which are highly conserved across DENV serotypes. CD4^+^ T cells aid in coordinating the immune response by producing cytokines and supporting B cell maturation and antibody production. During DENV infection, CD4^+^ T cells respond to epitopes on structural proteins such as the envelope (E), capsid (C), and NS1 proteins [53].

**Figure 7:**
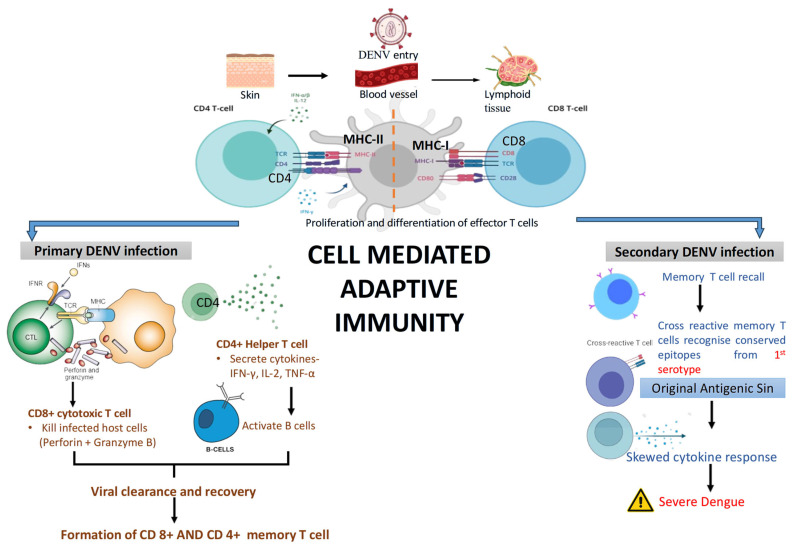
Cell-mediated adaptive immune response in DENV infection. During primary DENV infection, antigen-presenting cells activate CD4^+^ and CD8^+^ T cells. CD4^+^ helper T cells secrete cytokines (IFN-γ, IL-2, TNF-α) to support B cell activation, while CD8^+^ cytotoxic T cells kill infected host cells, aiding in viral clearance and memory T cell formation. In secondary infection, memory T cells are rapidly recalled. However, cross-reactive T cells may recognize conserved epitopes from the first infection (original antigenic sin), leading to a skewed cytokine response. This misdirected immune reaction contributes to severe dengue outcomes, including excessive inflammation and tissue damage.

As depicted in Figure 7, During primary DENV infection, antigen-presenting cells activate CD4^+^ and CD8^+^ T cells. CD4^+^ helper T cells secrete cytokines (IFN-γ, IL-2, TNF-α) to support B cell activation, while CD8^+^ cytotoxic T cells kill infected host cells, aiding in viral clearance and memory T cell formation. In secondary infection, memory T cells are rapidly recalled. However, cross-reactive T cells may recognize conserved epitopes from the first infection (original antigenic sin), leading to a skewed cytokine response. This misdirected immune reaction contributes to severe dengue outcomes, including excessive inflammation and tissue damage.

## 5. Host Immune Evasion Strategies of DENV

### 5.1. Movement of Immune Cells from Skin to Lymph Nodes: A Key Mechanism for DENV Immune Escape and Viral Spread

After a mosquito bite, DENV infects skin dendritic cells and macrophages, which transport it to lymph nodes and blood, leading to widespread infection of monocytes, macrophages, and endothelial cells in organs such as the spleen, liver, bone marrow, and kidneys. IL-1β and TNF stimulate this migration. This active replication, known as “active viremia,” begins 24–48 h before symptoms appear and can last for 10–12 days [54]. The migration of infected phagocytes to nearby lymph nodes triggers the activation of the adaptive immune response, boosts further DENV replication within both resident and newly arriving mononuclear phagocytes in the lymph nodes, and helps to spread infectious dengue virions into the bloodstream and circulating monocytes [27].

### 5.2. Innate Immune Evasion: Structural Hiding in the Host Cell, Inhibition of IFN-Production and Signalling

DENV employs various strategies to disrupt innate immune signalling, thereby weakening the host’s antiviral defences. To establish a productive infection, DENV must suppress the primary innate immune mechanism, which centres on the type I interferon (IFN) response and proinflammatory cytokine production. DENV evades the host’s innate immune response through different mechanisms, one of which involves the formation of vesicle packets (VPs) within the host’s endoplasmic reticulum [55]. These structures act as specialised compartments for storing viral proteins and metabolites, effectively shielding DENV from host antiviral defence mechanisms.

DENV evades IFN production by using its non-structural (NS) proteins to suppress the type I interferon response by interfering with its key sensors and messengers. NS4a disrupts the interaction between RIG-I, MDA-5 and the adaptor protein MAVS by binding to both the N-terminal CARD-like domain and the C-terminal transmembrane domain of MAVS, on mitochondria. In contrast, NS3 protein inhibits the translocation of RIG-I to MAVS. This interference results in the inhibition of IRF3 activation and a subsequent decrease in IFN production [55]. Another mechanism for blocking interferon production is the inhibition of the RIG-I/MAVS signalling pathway and the suppression of IRF3 phosphorylation by TBK1, mediated by the viral proteins NS2A and NS4B [56].

**Figure 8:**
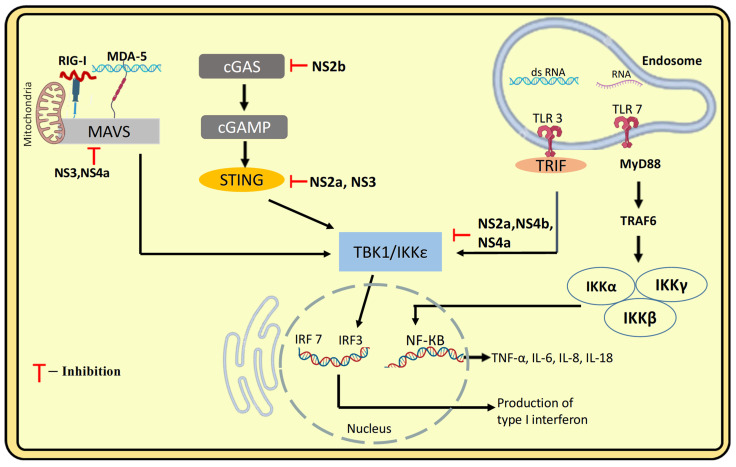
Strategies used by DENV to evade Innate immune responses. The figure illustrates how DENV interferes with the host innate immune signalling pathways that lead to type I interferon and proinflammatory cytokine production. In the cytoplasm, viral RNA is detected by RIG-I and MDA-5, which signal via MAVS on mitochondria. This pathway is targeted and inhibited by DENV proteins NS3 and NS4a. The cGAS–STING pathway, which senses cytosolic DNA and produces cGAMP to activate STING, is also blocked by NS2a, NS2b, and NS3. In endosomes, TLR3 recognizes double-stranded RNA and signals via TRIF, while TLR7 recognizes single-stranded RNA and signals via MyD88, activating TRAF6 and the IKK complex (IKKα, IKKβ, IKKγ). All these pathways converge on TBK1/IKKε, which phosphorylates IRF3, IRF7, and activates NF-κB, inducing type I interferons and cytokines such as TNF-α, IL-6, IL-8, and IL-18. DENV non-structural proteins (NS2a, NS4a, NS4b) inhibit these signalling steps, suppressing the antiviral response.

Another strategy of IFN suppression is the antibody dependent enhancement mechanism (ADE), where DENV antibody immune complexes engage Fc receptors (FcR) on target cells, which leads to downregulation of TLR-3, TLR-4, and TLR-7 [56]. Moreover, DENV blocks phosphorylation of STAT1 and promotes degradation of STAT2, leading to decreased Type I IFN signalling (Figure 8) [8].

### 5.3. Immune Evasion from Complement Response

The non-structural protein 1 (NS1) is secreted in large quantities during DENV infection and plays a central role in immune evasion of the complement response. NS1 protein by binding to complement protein C4 and activating C1S protease, leading to cleavage of C4 to C4b, inhibiting the classical and lectin pathways of the Complement Pathway [57]. Another mechanism of complement inactivation is that NS1 inhibits membrane attack complex (MAC) formation by binding to the complement regulator vitronectin (VN), resulting in the formation of an NS1–VN complex [58]. NS1 evades the complement response by competitively binding to mannose-binding lectin (MBL), thereby blocking MBL from recognising DENV and protecting the virus from being neutralised [37].

### 5.4. Evasion of Adaptive Immune Response

#### 5.4.1. Antigenic Variation to Evade Recognition by Neutralizing Antibodies

RNA viruses evolve rapidly due to the error-prone nature of their RNA-dependent RNA polymerases, which generate mutations during replication. These low-fidelity enzymes produce diverse viral quasispecies with varied antigenic epitopes, enabling the virus to escape host neutralizing antibodies. In DENV, mutations in domain III of the E protein have been shown to facilitate immune evasion from host neutralizing antibodies during DENV infection [59].

#### 5.4.2. Antibody Dependent Enhancement (ADE) of Infection

Patients may become infected with different DENV serotypes over the course of their lives. Studies have shown that individuals experiencing a secondary infection with a heterologous serotype face a greater likelihood of developing severe dengue than those with a primary infection, a risk often attributed to the mechanism of ADE. Studies found that patients with a prior dengue diagnosis had a higher risk of severe dengue (7.8%) compared to those with a first infection (3.8%), moreover the risk was greatest when the interval between primary and secondary infection exceeded two years, supporting evidence that cross-protection wanes after this period. A Cuban study, showed markedly higher severity when reinfection occurred after long intervals, highlighting the importance of timing between exposures, particularly with certain serotype sequences such as primary DENV-2 followed by secondary DENV-1 [60]. A prospective birth cohort study of Vietnamese infants reveal DENV rate in infants was 1.7 cases/100 person years, much lower than in older Vietnamese children. Almost 98% of women had prior DENV infection, and their infants were born with higher IgG titers than the mothers. Neutralizing maternal IgG decays quickly (undetectable in ~80% by 9 months), but binding (non-neutralizing) IgG persists longer. This creates a window (4–12 months) where virion-binding but non-neutralizing IgG may promote ADE. Plasma from 6-month-olds showed the greatest in vitro enhancement. Infection pattern reveals lower incidence in infants may be due to reduced mosquito exposure and possibly maternal antibody protection, which indicates, vaccination strategies should target older age groups, indirectly protecting infants [61,62]. Whereas in a Brazilian birth cohort, maternally transferred DENV specific IgG and neutralizing antibodies were higher in newborns than in mothers, especially from single serotype immune mothers. These antibodies declined rapidly, with neutralizing antibodies lost in >90% of infants by 4 months. Peak ADE occurred at 2 months and declined by 4 months, suggesting that the earlier waning of maternal antibodies may explain the lower frequency of severe dengue in Brazilian infants compared to Asian countries [63].

A separate study established an in vivo mouse model demonstrating ADE of dengue, analogous to infant infections with maternally transferred antibodies. Passive transfer of anti-dengue antibodies enhanced infection and lethal disease, showing hallmarks of severe dengue such as thrombocytopenia, vascular leakage, high cytokines, and elevated viral burden. While high antibody doses were protective, lower or cross-reactive doses enhanced disease, even when neutralizing in vitro. Importantly, a genetically modified anti-DENV antibody unable to bind Fcγ receptors prevented ADE and showed therapeutic potential [64].

Another study in pediatric population highlights that severe dengue is associated with accelerated avidity decay and lower long-term antibody avidity. A study of 42 secondary DENV2 cases showed that IgG avidity increased from acute to convalescent phase, declined by 3 months, and stabilized up to 18 months, similar cases seen in DENV3 infection. Over time, variability among individuals grew, with severe cases displaying significantly lower avidity and faster decay than mild cases. Regression confirmed accelerated decline in severe disease, and ROC analysis indicated that low avidity at later time-points may serve as a retrospective marker of severe dengue [60].

During secondary DENV infection, pre-existing antibodies from the first infection can enhance viral entry into Fc receptor bearing cells, leading to antibody-dependent enhancement (ADE) and more severe disease. This enhancement also facilitates immune evasion through two main mechanisms: (1) blocking antiviral signalling pathways (TLR and RIG-I/MDA-5) via negative regulators such as autophagy-related 5-authophagy-related 12 (Atg5–Atg12), selective androgen-receptor modulator (SARM), dihydroxyacetone kinase (DAK), thereby suppressing type I interferon production, and (2) shifting cytokine responses toward immunosuppression, with increased IL-10 and suppressor of cytokine signalling 3 (SOCS-3) mediated inhibition of the JAK-STAT pathway, while reducing IL-12 and IFN-γ. IL-10 also dampens T cell activity and promotes antibody production through B-cell activating factor (BAFF) and proliferation-inducing ligand (PRIL), further fueling ADE [59]. Additionally, antibodies against the prM protein can render immature DENV particles infectious during secondary infection, increasing viral load and potentially serving as a strategy for humoral immune evasion [65].

#### 5.4.3. Evasion by Antigen Presentation Blockade and T Cell Recognition

**Figure 9:**
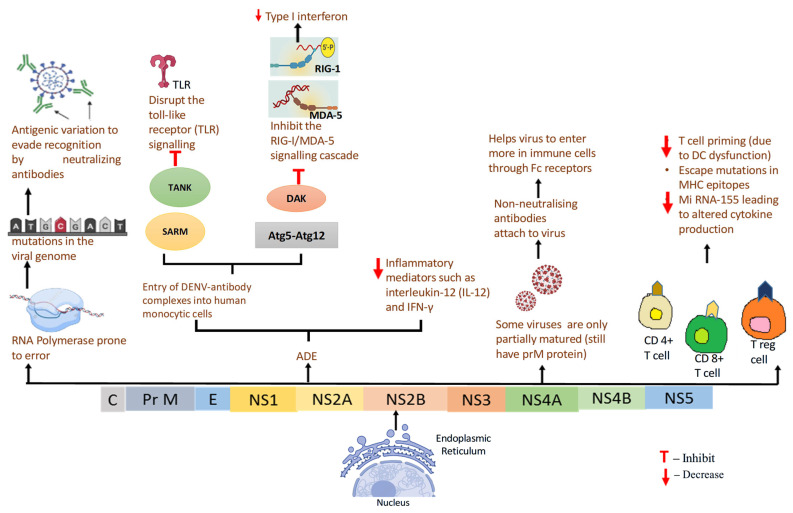
Strategies used by DENV to evade adaptive immune responses. DENV evades adaptive immunity through various mechanisms. Mutations arising from error-prone RNA polymerase result in antigenic variation, allowing for escape from neutralising antibodies. DENV exploits antibody-dependent enhancement (ADE), where non-neutralizing antibodies bind to the virus and promote its entry into immune cells via Fc receptors. Inside host cells, DENV suppresses interferon responses by disrupting Toll-like receptor (TLR) signalling pathways (via TANK, SARM) and inhibiting RIG-I/MDA-5 cascades through DAK and atg5-atg12. Some virions remain partially matured (retaining prM), contributing to enhanced infectivity. Viral mutations in MHC epitopes and dendritic cell dysfunction impair T cell priming. Also, miRNA-155 dysregulation alters cytokine signalling. Together, these steps interfere with CD4^+^, CD8^+^, and regulatory T cell responses, weakening adaptive immunity and increasing the risk of severe dengue disease.

Dendritic cells (DCs) link innate and adaptive immunity by presenting antigens, activating T cells, and secreting cytokines. DENV disrupts this process by inducing apoptosis in infected DCs and blocking their activation, while non-infected bystander DCs upregulate co-stimulatory molecules and remain functional. Infected DCs show reduced ability to prime CD4^+^ and CD8^+^ T cells, partly due to impaired IFN-α/β production, leading to weaker antiviral responses. Thus, by impairing antigen presentation and the functional capacity of infected DCs, DENV may evade immunity by limiting T cell activation [59]. Additionally, viral quasispecies generated during infection can alter T cell epitopes, reducing recognition by MHC molecules and T cell receptors. In heterologous secondary infections, this antigenic variation contributes to “original antigenic sin,” where memory T cells respond poorly to new serotypes, promoting inadequate immunity and severe disease [57,59]. Additional strategies used by the DENV to evade adaptive immune responses are illustrated in Figure 9.

## 6. Influence of Host Immunity and Genetic Factors on DENV Infection and Disease Severity

### 6.1. Effect of Host Immunity on the Pathogenesis of Dengue Infection

Both humoral and adaptive immune responses play a role in the development of DHF/DSS. In addition to the well-established ADE hypothesis, another proposed mechanism involves autoimmunity driven by molecular mimicry, which may also be crucial in the disease’s pathogenesis. Studies indicate that anti-nonstructural protein 1 (anti-NS1) antibodies cross-react with endothelial cells and platelets, resulting in vascular leakage and thrombocytopenia. The interaction of anti-NS1 antibodies with endothelial cells triggers the secretion of cytokines such as IL-6, IL-8, MCP-1, TNF-α, IL-1β, and GM-CSF, along with chemokines including RANTES (CCL5) and IP-10 (CXCL10), all of which contribute to endothelial activation, increased vascular permeability, and ultimately vascular leakage. [66,67]. Although the connection between anti-NS1 antibodies and platelets remains unclear, Th17 cells are increasingly recognised as key drivers of antibody-mediated autoimmunity. They may also contribute to the pathogenic effects observed with cross-reactive anti-NS1 antibodies in dengue. The principal cytokine produced by TH17 cells is IL-17, which can induce other cytokines and chemokines, including IL-6, IL-8, MCP-1, TNF-α, and IL-1β. These effector cytokines remain elevated in patients with severe dengue and play a significant role in vascular leakage. During DENV infection, disease outcomes often depend on a balance between Th1 and Th2 immune responses. When a Th2-biased response dominates, heightened levels of IL-10 and TGF-β suppress the Th1 pathway, a shift frequently associated with the development of severe dengue. Furthermore, regulatory T cells (Tregs) which also secrete IL-10 and TGF-β are believed to contribute to this immune shift by dampening Th1-mediated antiviral responses. Emerging evidence suggests that Tregs may release a prostaglandin-like factor that further inhibits IL-12 production, thereby worsening Th1 suppression during severe dengue [59].

### 6.2. Host Genetics Determinants and Susceptibility

#### 6.2.1. Major Histocompatibility Complex Genes

The Major Histocompatibility Complex (MHC) encodes both HLA class I (HLA-A, -B, -C) and HLA class II (HLA-DR, DQ, DP), which are responsible for presenting peptide antigens to host T-lymphocytes [68]. This interaction, as well as the anti-viral response, varies depending on the specific HLA allele involved. This HLA-specific allele influences the reactivity of DENV specific T cells. Some newly described alleles, HLA-B*53, HLA-B*13, and HLA-DQB1*302, have been linked to Dengue Haemorrhagic Fever (DHF) [69]. It has also been observed that human platelet antigen (HPA)-1a and HPA-2b alleles are associated with DHF, while HPA–1 heterozygotes are more likely to develop shock [70]. Additionally, the combination of the cytotoxic T-lymphocyte antigen 4 (CTLA-4) +49 G allele and the TGF-b1-509 CC genotype has been associated with increased susceptibility to DHF. HLA class III subregion, located within the class III region of the MHC, contains TNF, a crucial vasoactive immunomodulator produced by activated monocytes, which is known to be upregulated in DHF infection [71]. Polymorphism in the promoter region of the TNF-alpha gene, specifically the -308A allele, has been identified as a risk factor for the development of DHF in populations of South America. HLA alleles A*52 and DRB1*104 have been identified as protective factors against dengue infection, whereas alleles B*51, DQB1*0302, and the TNF-α–LTA haplotype are associated with increased susceptibility [72].

#### 6.2.2. Non-MHC Genes

Studies have also shown an association between polymorphic non-HLA genes and susceptibility to Dengue infection (DI). One such gene is FcγR, which is widely distributed across IgG subclasses and is responsible for ADE during DI [73]. This gene offers a protective function against DSS and DHF/DSS in populations of South-east Asia and South America. Another gene is the Vitamin D receptor (VDR), expressed on monocytes, activated B cells, and T cells. VDR polymorphism analysis revealed that the particular C allele provides resistance to DSS [72].

## 7. Recent Advances in Therapeutic Approaches and Vaccine Development

### 7.1. Symptomatic Management of Dengue

Due to the lack of specific antiviral treatments for dengue, current clinical management focuses on supportive care, with paracetamol (acetaminophen) being the recommended antipyretic according to WHO guidelines [74]. However, recent studies have raised concerns about the safety of paracetamol intake in dengue patients. A study reported a significantly higher incidence of elevated liver transaminases among patients receiving paracetamol compared to the placebo group. Since hepatic involvement is already a common complication in dengue, the use of paracetamol even at therapeutic doses may pose a risk of hepatotoxicity [75]. Moreover, the study found that paracetamol did not significantly reduce fever, pain, or hospitalisation duration, suggesting limited clinical benefit from its use. Additionally, intravenous (IV) fluid therapy is necessary for those exhibiting warning signs, persistent vomiting, rising haematocrit, or evidence of shock. According to WHO guidelines, 10 mL/kg of isotonic crystalloid solution should be administered over one hour for compensated shock, and 20 mL/kg over 15 min in cases of decompensated shock. Another characteristic feature of dengue infection is thrombocytopenia; current guidelines recommend avoiding NSAIDS and employing eltrombopag, a thrombopoietin receptor agonist, in dengue patients with moderate to severe thrombocytopenia [76].

### 7.2. Role of Therapeutics in Targeting the DENV

Direct-acting antivirals (DAAs) are a promising therapeutic approach because they target viral proteins vital for replication, such as NS3, NS4B, and NS5. A recent promising DAA is JNJ-A07, an NS4B inhibitor that blocks the interaction between NS4B and NS3 proteins, which are essential for viral replication, and has shown effectiveness against all four DENV serotypes in vitro and animal models. Ivermectin, although it exhibited NS5 nuclear transport inhibition in vitro and faster NS1 clearance in trials, did not lead to clinical improvement [77]. Doxycycline, an antibiotic, demonstrated anti-DENV activity in vitro and reduced inflammatory cytokines (IL-6, TNF) in early trials. Although controlled trials are ongoing to confirm its potential as a treatment for dengue infection, the results are still inconclusive. A few drugs, such as celgosivir and UV-4B, both alpha-glucosidase inhibitors, disrupt viral glycoprotein processing and target host cell processes [78]. Currently, zanamivir, a neuraminidase inhibitor, has shown the ability to prevent NS1-induced vascular leakage in vitro. However, its safety and potential benefits in patients with vascular permeability syndrome are still under assessment [79].

### 7.3. Vaccine Development

The development of an effective dengue vaccine has been recognised by the WHO as a vital global health priority, due to the significant public health impact and economic burden caused by the disease. However, progress in this field has been obstructed by several substantial challenges. One of the main difficulties is achieving a balanced immune response against all four DENV serotypes. This is complicated by antigenic competition, where the immune system tends to favour specific serotypes over others, resulting in an uneven protective response. Moreover, serious concerns regarding ADE exist, which present a substantial obstacle in developing a dengue vaccine, as insufficient or incomplete protection against any of the dengue serotypes may increase the risk of severe disease during subsequent infections [80]. Some of the leading vaccine candidates that have progressed the furthest in clinical development [70]. The list is given below:

CYD-TDV (Dengvaxia):

Developed by Sanofi Pasteur, this is a live attenuated tetravalent vaccine based on a yellow fever virus backbone. However, its use is limited to individuals who have previously had a dengue infection. This restriction exists due to the risk of antibody-dependent enhancement (ADE), which can result in more severe disease in people who receive the vaccine without prior dengue exposure [81].

TAK-003 (Qdenga):

Developed by Takeda, this vaccine is based on a live attenuated DENV-2 strain, which acts as the foundation for all four serotypes. It has demonstrated promising efficacy against DENV-2 infection and lower efficacy against other serotypes [82].

TV003/TV005:

Developed by the National Institute of Allergy and Infectious Diseases (NIAID), this is a live attenuated tetravalent vaccine. A single dose of the TV003 or TV005 vaccine elicited an immune response (seroconversion) against all four DENV serotypes in 74–92% of individuals who had not previously been exposed to flaviviruses, with TV005 demonstrating slightly higher effectiveness at 90%. Furthermore, this initial dose offered strong, nearly complete protection, significantly reducing the immune response to a second dose administered 6 to 12 months later, implying that the first dose had already induced near-sterilising immunity [83].

## 8. Current Challenges and Future Directions in Dengue Therapy and Vaccine Development

Although progress has been made, significant gaps still hinder the effectiveness of dengue prevention and treatment strategies. From a therapeutic perspective, many existing guidelines by the WHO are available, particularly those related to fluid therapy. Still, they lack strong support from randomised controlled trials (RCTs) [84]. Ongoing research is exploring various approaches, including modified fluid regimens, the use of albumin, and innovative alternative treatments to manage better dengue-related complications such as shock and low platelet count [85]. There remains an urgent need for more robust, evidence-based therapies to address key issues like liver damage, bleeding tendencies, and fluid overload. In vaccine development, achieving a well-balanced immune response against all four dengue serotypes remains a significant challenge, as current vaccines often demonstrate uneven efficacy. Safety concerns in individuals without prior dengue exposure persist due to the potential for ADE, which may increase the severity of subsequent infections. Ensuring long-term immunity is also problematic, especially for vaccines like TAK-003, where protective effects may diminish over time, possibly requiring booster doses [75]. Additionally, there is increasing focus on developing vaccines that stimulate T-cell responses, potentially by incorporating non-structural proteins from each serotype [16].

## 9. Proteomic and Transcriptomic Insights into Early Host Biomarkers of Severe Dengue

### 9.1. Proteomics Insights

About 20–25% of DENV infections become symptomatic, ranging from self-limiting fever to shock [86]. However, not all dengue patients with warning signs progress to severe dengue; only 5% develop severe disease. To elucidate the host factors associated with severe dengue, several studies have employed proteomics-based approaches. One such observational study, conducted as part of the DENIM cohort in Vietnam, analysed plasma samples collected 6–48 h before the onset of severe symptoms or defervescence. The study utilised individual iTRAQ labelling among eight subjects, enabling relative quantification of plasma proteins [87]. The analysis identified five proteins as overexpressed and fourteen as underexpressed, all of which were involved in key biological processes, including blood coagulation, vascular regulation, cellular transport, and immune responses. These proteins were associated with functions including protease activity, protease inhibition, transaminase function, membrane trafficking, cell adhesion, and signal transduction. From this dataset, two proteins Angiotensinogen and Antithrombin-III were selected for further validation based on their functional relevance. Their differential expression was subsequently confirmed using Western blot analysis, reinforcing their potential roles as host biomarkers or contributors to the pathophysiology of severe dengue. Another study, conducted on plasma using TMT-based quantitative proteomics, found differentially expressed proteins in DF patients before they develop into DHF. These proteins are involved in a variety of biological processes, including the proteasome pathway, alanine, aspartate, and glutamate metabolism, as well as arginine biosynthesis. Thus, PLAT, LAMB2, F9, VCAM1, FGL1, MFAP4, and GLUL could be considered as potential markers for predicting DHF since the levels of these proteins vary between DF and DHF [88].

### 9.2. Transcriptomics Insights

Further transcriptomics-based studies were also carried out, using a dual RNA-sequencing (RNA-Seq) approach on serum samples from individuals infected with dengue to explore early transcriptional changes. This analysis revealed distinct gene expression patterns that distinguished patients with high viral RNA levels from those with low viral loads. Functional interpretation indicated that two major gene sets were significantly upregulated in the high viral load group: one related to antiviral responses (IFIT3, RSAD2, SAT1) and another associated with vascular dysfunction (TNFS10, CXCL8). A closer look at these gene profiles suggests a dual host response, while the antiviral genes may help produce a milder disease outcome, the increased expression of vascular-related genes could signal progression towards severe disease [89]. This integrated genomics approach considers both host and pathogen roles to offer crucial insights into host immune responses and clinical outcomes. Additionally, liver transcriptomic studies in mouse models showed that DENV infection causes significant differential gene expression (IL10, SOCS3, IL1RN, IL6, VCAM1, IL1R1, CCL4, GATA3, LBP, ICAM1, and PF4), which participate in signalling pathways such as leukocyte trans-endothelial migration, complement and coagulation cascades, cytokine-cytokine receptor interactions, and viral protein interactions with cytokine and cytokine receptors [90].

## 10. Discussion

The immune response against DENV involves a dynamic interplay between the innate and adaptive arms of immunity, which collectively determine disease outcome. While innate mechanisms such as interferon signalling, NK cell activation, and cytokine production are crucial for early control, their dysregulation often drives immunopathology. Similarly, adaptive immunity can either limit infection through neutralizing antibodies and cytotoxic T cells or exacerbate disease through cross-reactive, non-neutralizing antibodies and imbalanced T cell responses.

DENV exploits multiple evasion strategies, including suppression of interferon pathways, impaired antigen presentation, and manipulation of autophagy and apoptosis, to sustain replication and dissemination. The role of multifunctional non-structural protein 1 (NS1) in disrupting vascular integrity and complement regulation further emphasizes how viral proteins directly contribute to severe clinical outcomes.

Severe dengue is strongly linked to secondary heterotypic infection, where prior exposure to one serotype predisposes individuals to worse outcomes with another. This is primarily due to ADE, where sub-neutralizing antibodies promote viral entry into Fc receptor–bearing cells, increasing viral load and immune activation. In parallel, cross-reactive memory T cells may drive excessive cytokine release of “original antigenic sin”, together amplifying immunopathology and explaining the higher severity seen in secondary infections.

Current management remains largely supportive, with fluid therapy as the cornerstone of treatment. Direct-acting antivirals and host-targeted therapies are under evaluation, but translation to clinical practice is still limited. Vaccine development continues to face challenges related to serotype coverage and the risk of ADE, although newer tetravalent candidates show encouraging immunogenicity. Moreover, in recent years, the identification of host and viral biomarkers has enhanced our understanding of DENV pathogenesis and provided potential tools for early severity prediction. Transcriptomic and proteomic profiling have further revealed signatures that distinguish between uncomplicated and severe cases, offering prospects for patient stratification and targeted interventions.

A balanced immune response capable of controlling viral replication while avoiding immune-mediated damage is essential for favourable dengue outcomes. The integration of biomarker-guided severity assessment, immune-modulating therapeutics, DAAs, and safe tetravalent vaccines represents a comprehensive path forward in dengue control.

## Data Availability

No new data were created or analyzed in this study.

## Abbreviations

ADE	Antibody-dependent enhancement
APRIL	A Proliferation-inducing ligand
Argo	Argonaute
CMI	Cell-mediated immunity
CTLA-4	Cytotoxic T-lymphocyte antigen 4
CTLs	Cytotoxic T Lymphocytes
CXCL8	C-X-C motif chemokine ligand 8
DAAs	Direct Acting Antivirals
DAK	Dihydroxyacetone kinase
DAMPs	Damage-associated molecular patterns
DC-SIGN	Dendritic cell-specific intercellular adhesion molecule-3-grabbing non-integrin
DENV	Dengue Virus
DHF	Dengue haemorrhagic fever
DSS	Dengue shock syndrome
FGL1	Fibrinogen-like protein 1
GATA3	GATA binding protein 3
GLUL	Glutamate-ammonia ligase
ICAM1	Intercellular Adhesion Molecule 1
IFIT3	Interferon-Induced Protein with Tetratricopeptide Repeats 3.
IL-6	Interleukin-6
IL1R1	Interleukin-1 Receptor, Type I
IL1RN	Interleukin-1 Receptor Antagonist
ISGs	Induces interferon-stimulated genes
LAMB2	laminin subunit beta-2
MAC	Membrane attack complex
MBL	Mannose-binding lectin pathway
MCP-1	Monocyte Chemoattractant Protein-1
MDA-5	Melanoma Differentiation-Associated gene 5
MFAP4	Microfibril-associated protein 4
MICB	Metabolic, Circulatory, Immune, and microbial microenvironment
moDCs	Monocyte-Derived Dendritic Cells
NIAID	National Institute of Allergy and Infectious Diseases
NK Cells	Natural Killer Cells
OAS	Original antigenic sin
PAMPs	Pathogen-associated molecular patterns
pDCs	Plasmacytoid dendritic cells
PF4	Platelet Factor 4
PLAT	Tissue-type Plasminogen Activator
PRRs	Pattern recognition receptors
RIG-I	Retinoic acid-inducible gene I
RISC	RNA-induced silencing complex
RLRs	RIG-like receptors
RSAD2	Radical S-adenosyl methionine domain containing 2
SARM	Selective androgen-receptor modulator
SOCS-3	Suppressor of cytokine signalling 3
STAT2	Signal Transducer and Activator of Transcription 2
TBK1	TANK-binding kinase 1
TLRs	Toll-like Receptors
TNF-α	Tumor necrosis factor alpha
TNFS10	Tumor Necrosis Factor (Ligand) Superfamily, Member 10
TRIF	TIR-domain-containing adapter-inducing interferon-β
VCAM1	Vascular Cell Adhesion Molecule 1
VDR	Vitamin D receptor
VN	Vitronectin
VPs	Vesicle packets
WHO	World Health Organization
